# Systematic review and meta-analysis of tocilizumab in persons with coronavirus disease-2019 (COVID-19)

**DOI:** 10.1038/s41375-021-01264-8

**Published:** 2021-05-17

**Authors:** Chong-xiang Chen, Fang Hu, Jin Wei, Le-tao Yuan, Tian-meng Wen, Robert Peter Gale, Yang Liang

**Affiliations:** 1 Department of Hematologic Oncology, State Key Laboratory of Oncology in South China, Collaborative Innovation Center for Cancer Medicine, Sun Yat-sen University Cancer Center, Guangzhou, China; 2 State Key Laboratory of Respiratory Disease, Guangzhou Institute of Respiratory Health, First Affiliated Hospital of Guangzhou Medical University, Guangzhou, Guangdong Province China; 3 Department of ICU, State Key Laboratory of Oncology in South China, Collaborative Innovation Center for Cancer Medicine, Sun Yat-sen University Cancer Center, Guangzhou, China; 4 Department of Hematology, Affiliated Hospital of North Sichuan Medical College, Nanchong, Sichuan China; 5 School of Public Health, Sun Yat-sen University, Guangzhou, China; 6 Department of Immunology and Inflammation, Haematology Research Centre, Imperial College London, London, UK

**Keywords:** Infectious diseases, Immunotherapy

## Abstract

We performed a meta-analysis to determine safety and efficacy of tocilizumab in persons with coronavirus disease-2019 (COVID-19). We searched PubMed, Web of Science and Medline using Boolean operators for studies with the terms coronavirus OR COVID-19 OR 2019-nCoV OR SARS-CoV-2 AND tocilizumab. Review Manager 5.4 was used to analyze data and the modified Newcastle–Ottawa and Jadad scales for quality assessment. We identified 32 studies in 11,487 subjects including three randomized trials and 29 cohort studies with a comparator cohort, including historical controls (*N* = 5), a matched cohort (*N* = 12), or concurrent controls (*N* = 12). Overall, tocilizumab decreased risk of death (Relative Risk [RR] = 0.74; 95% confidence interval [CI], 0.59, 0.93; *P* = 0.008; *I*^*2*^ = 80%) but not of surrogate endpoints including ICU admission (RR = 1.40 [0.64,3.06]; *P* = 0.4; *I*^*2*^ = 88%), invasive mechanical ventilation (RR = 0.83 [0.57,1.22]; *P* = 0.34; *I*^*2*^ = 65%) or secondary infections (RR = 1.30 [0.97,1.74]; *P* = 0.08; *I*^*2*^ = 65%) and increased interval of hospitalization of subjects discharged alive(mean difference [MD] = 2 days [<1, 4 days]; *P* = 0.006; *I*^*2*^ = 0). RRs of death in studies with historical controls (RR = 0.28 [0.16,0.49; *P* < 0.001]; *I*^*2*^ = 62%) or a matched cohort (RR = 0.68 [0.53, 0.87]; *P* = 0.002; *I*^*2*^ = 42%) were decreased. In contrast, RRs of death in studies with a concurrent control (RR = 1.10 [0.77, 1.56]; *P* = 0.60; *I*^*2*^ = 85%) or randomized (RR = 1.18 [0.57,2.44]; *P* = 0.66; *I*^*2*^ = 0) were not decreased. A reduced risk of death was not confirmed in our analyses which questions safety and efficacy of tocilizumab in persons with COVID-19.

## Introduction

Infection with severe acute respiratory syndrome coronavirus-2 (SARS-CoV-2) causes coronavirus disease-2019 (COVID-19), an important pathogenetic component of which is cytokine release syndrome (CRS). CRS is mediated, at least in part, by interleukin-6 (IL-6). Tocilizumab, a humanized monoclonal antibody, selectively targets the interleukin-6 receptor (IL-6r) [1, 2] and is reported safe and effective in other settings such as after chimeric antigen receptor (CAR)-T-cell therapy, rheumatoid arthritis, and giant cell arteritis [3, 4].

Data from 11 uncontrolled studies [5–16] and most prior meta-analyses [17–24] claim tocilizumab is safe and effective in persons with COVID-19. However, data from three recent randomized controlled trials (RCTs) question this conclusion [25–27].

We conducted a systematic review and meta-analysis of 32 studies of safety and efficacy of tocilizumab in persons with COVID-19 which had a comparator cohort. Overall, we found tocilizumab decreased risk of death but not rates of intensive care unit(ICU) admission, invasive mechanical ventilation, secondary infections, and increased interval of hospitalization in persons discharged alive. However, a reduced risk of death was not confirmed in our analyses of studies with concurrent controls nor randomized trials. These data question safety and efficacy of tocilizumab in persons with COVID-19.

## Methods

### Search strategy and selection criteria

**Fig. 1:**
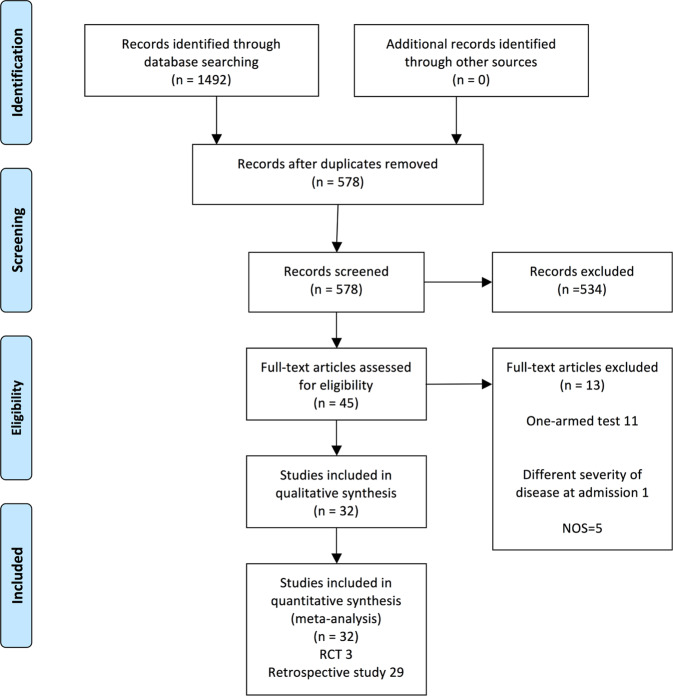
Flow diagram. Citation review process.

PubMed, Web of Science and Medline were searched using Boolean operators for studies with the terms coronavirus OR COVID-19 OR 2019-nCoV OR SARS-CoV-2 AND tocilizumab. Start and stop dates were 2020/1/1 and 2020/10/27. Two investigators independently reviewed abstracts of identified citations and selected articles for full review. Discordances were resolved by a third reviewer. Results were also manually searched and reviewed. We found 1492 articles excluding 914 duplicates. After further review we focused on 32 studies, 29 non-randomized comparator studies, and three RCTs [1, 2, 25–52]. A flow diagram of the search strategy and article selection is displayed in Fig. 1. Review Manager 5.4 was used to analyze data and the modified Newcastle-Ottawa score (NOS) and Jadad scale for quality assessment.

#### Inclusion and exclusion criteria

Inclusion criteria included English language reports of clinical trials and observational studies with a comparator cohort and with outcomes reporting, but not limited to, survival. Reviews and case reports were excluded as were studies with a NOS < 6.

#### Data extraction

For each selected article we extracted (1) first author, (2) publication year, (3) country, (4) study-design, (5) number of subjects, (6) comparator cohort, (7) baseline subject clinical and laboratory co-variates, (8) details of tocilizumab use, (9) concurrent interventions, and (10) outcomes, including: (1) survival, (2) rates of ICU admission, (3) invasive mechanical ventilation, (4) secondary infection, and (5) interval of hospitalization. The study used anonymized, published data with no requirement for Ethics Committee approval. (Table 1; Supplement Table 1)

**Table 1:** Included Studies.

Ref	Study-type/control cohort	NOS	Deaths (Tocilizumab vs. control)
[2]	Matched	9	17/64 vs. 24/64
[1]	Historical	8	5/32 vs. 11/33
[29]	Matched	8	102/210 vs. 256/420
[49]	Matched	9	23/106 vs. 61/138
[28]	Matched	8	3/22 vs. 2/22
[31]	Concurrent	8	5/21 vs. 19/91
[30]	Historical	8	2/62 vs. 11/23
[36]	Concurrent	7	44/132 vs. 97/475
[35]	Historical	7	13/179 vs. 73/365
[33]	Matched	9	5/29 vs. 19/58
[34]	Concurrent	7	14/59 vs. 16/87
[38]	Concurrent	7	3/28 vs. 2/23
[40]	Matched	6	8/30 vs. 66/176
[41]	Concurrent	6	61/260 vs. 120/969
[42]	Concurrent	6	2/76 vs. 8/62
[43]	Historical	6	10/41 vs. 20/38
[50]	Matched	8	15/74 vs. 59/148
[52]	Concurrent	6	9/92 vs. 1/89
[48]	Concurrent	6	43/96 vs. 55/97
[32]	Historical	6	7/90 vs. 34/68
[58]	Concurrent	7	NA
[46]	Matched	9	2/40 vs. 12/40
[47]	Matched	9	0/10 vs. 1/10
[45]	Matched	9	12/29 vs. 8/29
[44]	Matched	9	2/20 vs. 3/40
[39]	Concurrent	7	19/54 vs. 11/57
[37]	Concurrent	7	119/433 vs. 1295/3491
[51]	Concurrent	7	14/78 vs. 27/76
[57]	Matched	7	NA
[26]	RCT	Jadad 4	2/60 vs. 1/66
[27]	RCT	Jadad 4	7/63 vs. 8/67
[25]	RCT	Jadad 5	9/161 vs. 3/81

*NOS* Newcastle–Ottawa scale, *RCT* randomized controlled trial, *NA* not available.

#### Risk of bias assessment

Risk of bias was assessed using the Jadad scale in four domains: (1) random sequence generation, (2) allocation concealment, (3) blinding of participants, and (4) complete reporting of withdrawals and dropouts [53]. Methodological quality of comparator studies was assessed using the modified Newcastle–Ottawa scale (NOS) [54, 55] consisting of three domains: (1) subject selection, (2) comparability of the study groups, and (3) outcomes assessment. A score of 0–9 was allocated to each relevant study. Observational studies with a NOS score <6 (*N* = 1) were excluded [56].

#### Statistics

We pooled data and utilized Relative Risks (RRs) and Confidence Intervals (CIs) to describe dichotomous outcomes, including risk of death, ICU admission, invasive mechanical ventilation, secondary infection . We used mean difference (MD) and CIs for continuous outcomes including interval of hospitalization. We grouped the cohort studies into unmatched historical controls and concurrent controls, matched and unmatched or subgroup analyses. A fixed-effects model was used when *I*^*2*^ ≤ 50% and the Cochran Q statistic *P* > 0.1 and a random-effects model when *I*^*2*^ > 50% and Q statistic *P* ≤ 0.1. Funnel plots were used to screen for potential publication bias. Statistical analyses were carried out with Review Manager 5.4 (Cochrane Collaboration)

## Results

We included 11,487 subjects from 29 studies (NOS scores 6–9) with a comparator cohort including historical controls (*N* = 5) [1, 30, 32, 35, 43], a matched cohort (*N* = 12) [2, 28, 29, 33, 40, 44–47, 49, 50, 57] or concurrent controls (*N* = 12) [31, 34, 36–39, 41, 42, 48, 51, 52, 58] and three randomized controlled trials (Jadad scales 4–5). We excluded some studies because they were uncontrolled (*N* = 11) [5–16] and/or different study-entry severities of COVID-19 between treated subjects and controls (*N* = 1) [59]. Two studies reported only a composite endpoint (ICU admission, use of mechanical ventilation or death) [57, 58]. These were included in the systemic review but not used to estimate RRs for specific endpoints.

### Survival

**Fig. 2:**
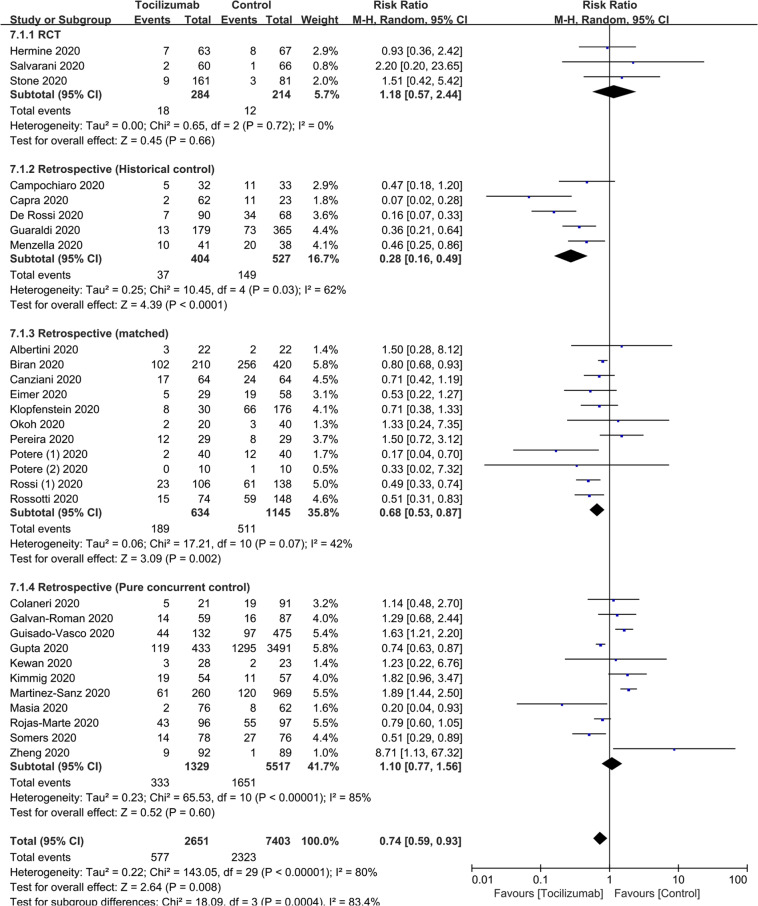
The effect of tocilizumab on survival. Risk of death.

To test the impact of tocilizumab on survival we included 30 studies, three RCTs and 27 other comparator studies of 10,054 subjects. [1, 2, 25–52] Relative Risk (RR) of death = 0.74 (95% Confidence Interval [CI], 0.59, 0.93; *P* = 0.008; *I*^*2*^ = 80%). Studies with historical controls (RR = 0.28 [0.16, 0.49]; *P* < 0.001; *I*^*2*^ = 62%) or with an otherwise matched cohort (RR = 0.68 [0.53, 0.87]; *P* = 0.002; *I*^*2*^ = 42%) reported significant survival improvement. In contrast, RRs of death in studies with concurrent controls (RR = 1.10 (0.77, 1.56; *P* = 0.60; *I*^*2*^ = 85%; Table 2, Fig. 2) and randomized trials (RR = 1.18 (0.57, 2.44; *P* = 0.66; *I*^*2*^ = 0; Table 2, Fig. 2) showed no significant improvement in survival.

**Table 2:** Risk of Survival and Surrogate Clinical Endpoints.

Ref	Risk of death (RR [95%CI])	Risk of ICU admission (RR [95%CI])	Risk of invasive mechanical ventilation (RR [95%CI])	Risk of secondary infection (RR [95%CI])
Observational (historical control)	0.28 [0.16, 0.49]	NA	0.74 [0.40, 1.37]	2.18 [0.68, 6.98]
Observational (matched)	0.68 [0.53, 0.87]	2.25 [1.17, 4.33]	0.77 [0.33, 1.80]	1.12 [0.77, 1.62]
Observational (concurrent control)	1.10 [0.77, 1.56]	1.91 [0.47, 7.69]	NA	1.27 [0.85, 1.89]
Randomized controlled trials	1.18 [0.57, 2.44]	0.80 [0.49, 1.33]	0.92 [0.21, 4.10]	0.26 [0.03, 2.28]
Total events	0.74 [0.59, 0.93]	1.40 [0.64, 3.06]	0.83 [0.57, 1.22]	1.30 [0.97, 1.74]

*RR* relative risk, *NA* not available.

### Surrogate clinical endpoints

**Fig. 3:**
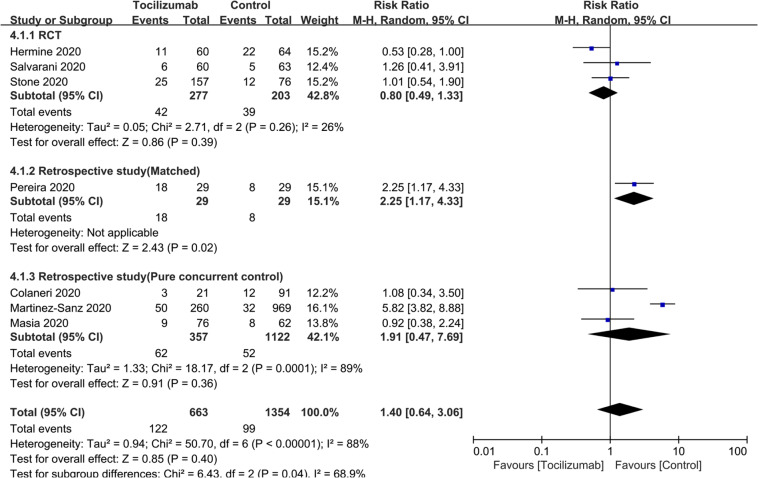
The impact of tocilizumab on ICU admission. Risk of ICU admission.

**Fig. 4:**
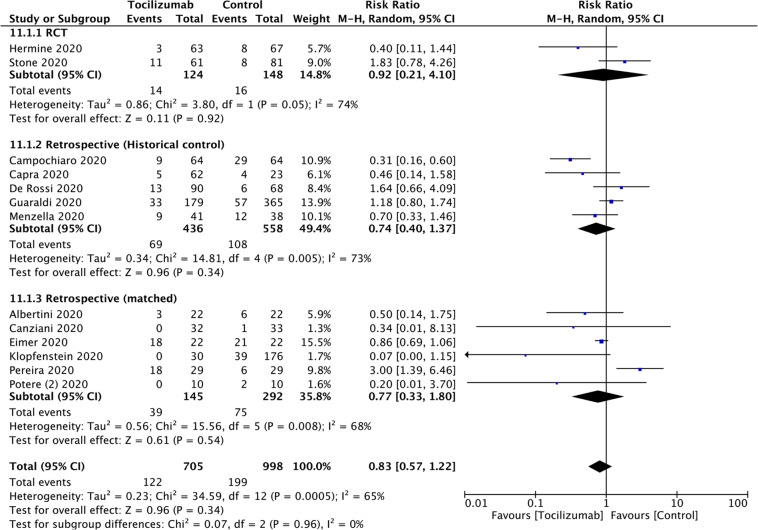
The association of tocilizumab and invasive mechanical ventilation. Risk of invasive mechanical ventilation.

**Fig. 5:**
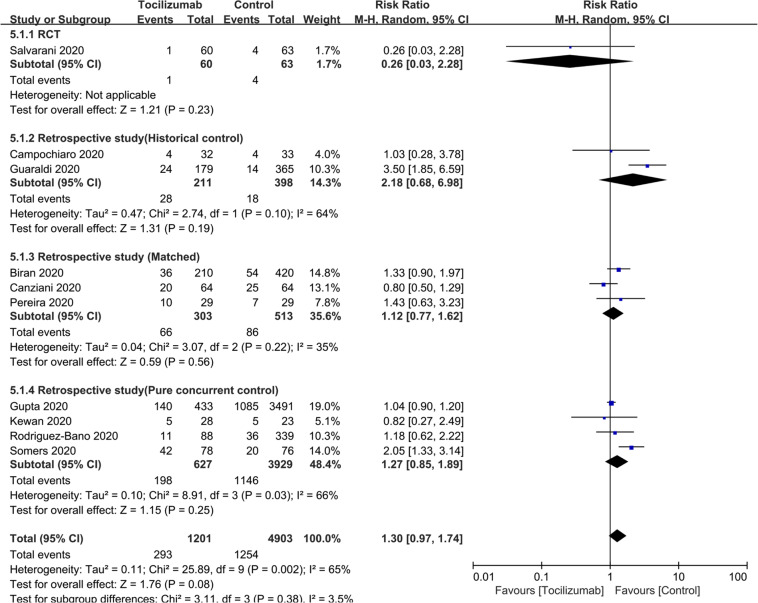
The correlation between tocilizumab and secondary infection. Risk of secondary infection.

To test the efficacy of tocilizumab on rate of ICU admission we included seven studies [25–27, 31, 41, 42, 45] of 2017 subjects. RR = 1.40 (0.64, 3.06; *P* = 0.4; *I*^*2*^ = 88%). RR for RCTs and for studies with concurrent controls were RR = 0.80 (0.49, 1.33; *P* = 0.39; *I*^*2*^ = 26%) and RR = 1.91 (0.47, 7.69; *P* = 0.36; *I*^*2*^ = 89%; Table 2; Fig. 3). (There was only one study with a matched cohort and no study with a historical control). To test the efficacy of tocilizumab on rate of invasive mechanical ventilation we included 13 studies [1, 2, 25, 27, 28, 30, 32, 33, 35, 40, 43, 45, 47, 56] of 1703 subjects. RR = 0.83 (0.57, 1.22; *P* = 0.34; *I*^*2*^ = 65%). RRs of studies with historical controls (RR = 0.74 [0.40, 1.37]; *P* = 0.34; *I*^*2*^ = 73%), those with a matched cohort (RR = 0.77 [0.33, 1.80]; *P* = 0.54; *I*^*2*^ = 68%) and RCTs (RR = 0.92 [0.21, 4.10]; *P* = 0.92; *I*^*2*^ = 74) are indicated. (There were no studies with concurrent controls; Table 2; Fig. 4). To test the effect tocilizumab on rate of secondary infections we included 10 studies [1, 2, 26, 29, 35, 37, 38, 45, 51, 58] of 5495 subjects. RR = 1.30 (0.97, 1.74; *P* = 0.08; *I*^*2*^ = 65%). RR for studies with a historical controls (RR = 2.18 [0.68, 6.98]; *P* = 0.19; *I*^*2*^ = 64%), those with a matched cohort (RR = 1.12 [0.77, 1.62]; *P* = 0.56; *I*^*2*^ = 35%) and those with concurrent controls (RR = 1.27 [0.85, 1.89]; *P* = 0.25; *I*^*2*^ = 66%) were indicated in Fig. 5. (There was only one RCT). To test the impact of tocilizumab on hospitalization interval we included seven studies [30, 32, 38, 40, 42, 51, 52] of 1041 subjects. Mean difference in subjects discharged from hospital was 2 days (<1, 4 days; *P* = 0.006; *I*^2^ = 0; Supplement Fig. 1).

### Publication bias

**Fig. 6:**
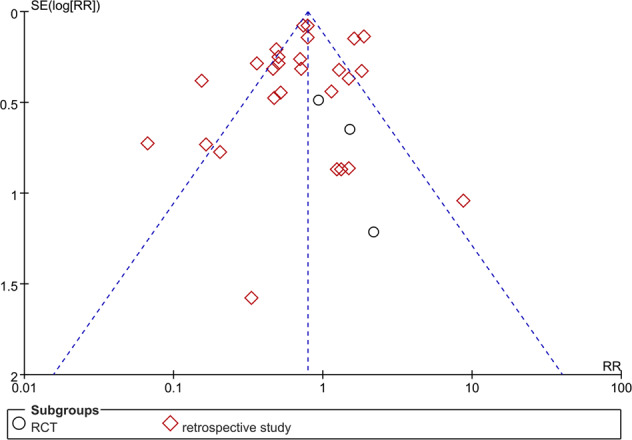
Funnel plot. Risk of publication bias.

Potential for publication bias is shown in Fig. 6. We found potential publication bias in studies of death in subjects receiving or not receiving tocilizumab with some studies falling outside the 95% CI of the funnel plot. There was publication bias in studies included in the meta-analysis.

## Discussion

Increased concentrations of inflammatory cytokines (IL-6, granulocyte-macrophage colony stimulating factor (GM-CSF) and tumor necrosis factor-a (TNF-a) are reported in persons with COVID-19 [60, 61]. IL-6 is produced by diverse immune cells and implicated in development of acute respiratory distress syndrome (ARDS) and CRS [62, 63]. Some data suggest increased IL-6 concentrations correlate with risk of death [61, 64]. Several meta-analyses claim tocilizumab is safe and effective in COVID-19 [17, 21, 23, 24]. Most studies we include gave tocilizumab to subjects with evidence of inflammation including a CRP concentration >100 mg/L, a ferritin concentration >900 ng/ml and/or a D-dimer concentration >1500 ug/L [1].

Evaluating all 32 studies we found tocilizumab reduced risk of death but not several surrogate endpoints, including ICU admission, invasive mechanical ventilation, and secondary infections. Hospitalization interval was significantly increased. However, in our analysis of RCTs and studies with a concurrent control cohort we could not confirm a decreased risk of death. This conclusion differs from most prior meta-analyses [17–24] which failed to analyze outcomes by study-design (Table 3). A recent meta-analysis concluded an association between tocilizumab and lower mortality by low certainty evidence from cohort studies [22]. Our data contradict this assumption. Two recent analyses which included only studies with a comparator cohort support our conclusion [65, 66]. Also, Mao and colleagues reported use of tocilizumab did not decrease risk of death possibly because of an increased risk of secondary bacterial infections [67].

**Table 3:** Previous meta-analyses.

Ref	*N* studies	*N* subjects	Studies included	RR or OR of death (95% CI)
[19]	13	766	2 historical controls 1 matched control 4 concurrent controls 6 no control	RR = 0.56 [0.34, 0.92]
[17]	23	6279	4 historical controls 5 matched controls 14 concurrent controls	RD = −0.06 [−0.12, −0.01]
[18]	16	3641	4 historical controls 3 matched controls 9 concurrent controls	OR = 0.57 [0.36–0.92]
[20]	7	592	1 historical control 3 matched controls 3 concurrent controls	RR = 0.62, [0.31,1.22]
[21]	10	1358	3 historical controls 1 matched control 6 concurrent controls	RR = 0.27 [0.12, 0.59]
[22]	23	11346	3 historical controls 6 matched controls 8 concurrent controls 1 no control 5 RCTs	RR for cohort studies = 0.58 [0.51–0.66] RR for RCTs = 1.09 [0.80,1.49]
[23]	10	1675	3 historical controls 2 matched controls 5 concurrent controls	OR = 0.47 [0.36, 0.60]
[24]	19	2285	4 historical controls 4 matched controls 5 concurrent controls 6 no controls	OR = 0.44 [0.36, 0.55]

*RR* relative risk, *OR* odds ratios, *RD* risk differences, *RCTs* randomized controlled trials.

There are important limitations to our study. Firstly, subjects were heterogeneous in COVID-19 severity although most had severe to critical COVID-19. Secondly, many studies were observational and lacked an appropriate control cohort. We tried to overcome potential biases in these studies by analyzing outcomes by study-type.

In conclusion, tocilizumab decreased risk of death but not rates of surrogate endpoints including ICU admission, invasive mechanical ventilation, secondary infections and did significantly alter interval of hospitalization. A reduced risk of death was not confirmed in our meta-analysis of randomized trials or studies with a concurrent control cohort. These data question safety and efficacy of tocilizumab in persons with COVID-19.

## Supplementary information

Supplementary Legends

Supplemental Figure 1

Supplemental Table 1
