# Supplementary figures and images for: Systematic review and meta-analysis of tocilizumab in persons with coronavirus disease-2019 (COVID-19)
Source: Leukemia. 2021 May 17;35(6):1661–70. doi: 10.1038/s41375-021-01264-8 (PMC8127467; doi:10.1038/s41375-021-01264-8)

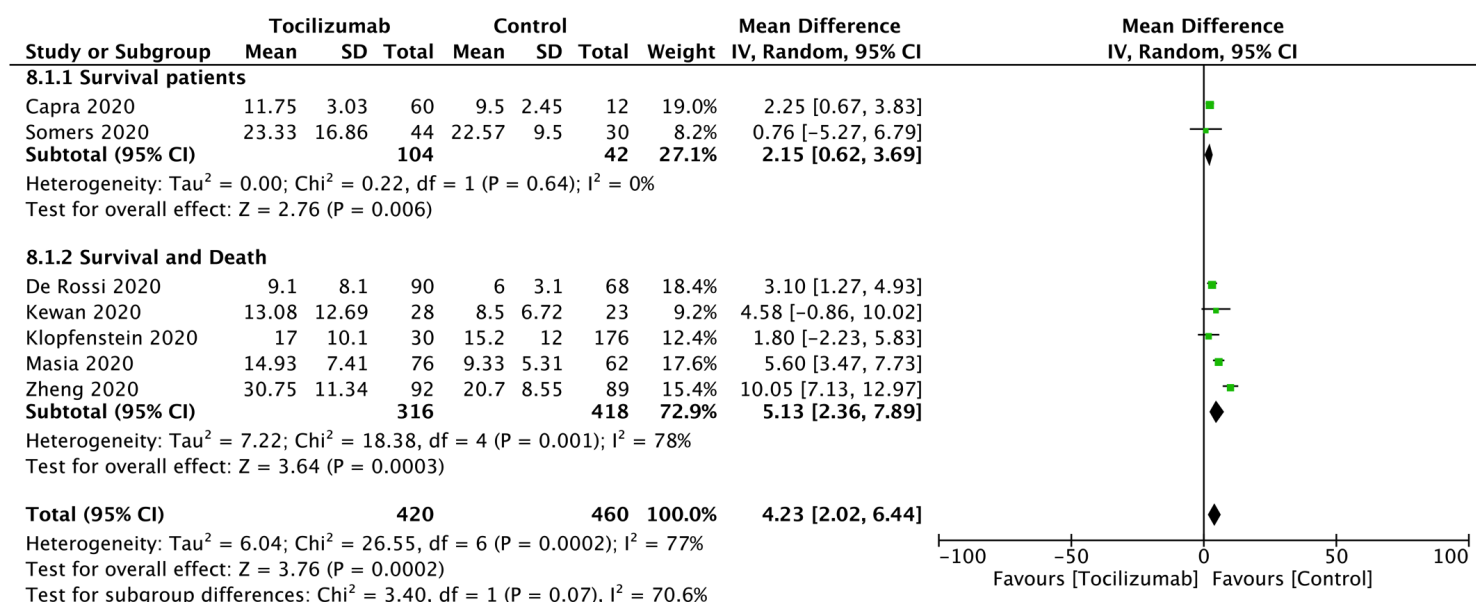

Supplement: Supplementary file 2 — Supplemental Figure 1 [file 41375_2021_1264_MOESM2_ESM.pdf]
