# Supplementary material for: Systematic review and meta-analysis of tocilizumab in persons with coronavirus disease-2019 (COVID-19)
Source: Leukemia. 2021 May 17;35(6):1661–70. doi: 10.1038/s41375-021-01264-8 (PMC8127467; doi:10.1038/s41375-021-01264-8)
Supplement: Supplementary file 3 — Supplemental Table 1 [file 41375_2021_1264_MOESM3_ESM.docx]

**Supplement Table 1. Characteristics of studies included in the meta-analysis**

| Ref | Country | Dates | Participants | Enrolled patients | Timing of using tocilizumab | Tocilizumab usage (Dose and schedule) |
| --- | --- | --- | --- | --- | --- | --- |
| [2] | Italy | 2020-2-23 to 2020-5-29 | 2 centers | Adult patients with COVID-19 and need respiratory support, and elevated CRP | Clinical worsening in the previous 24h; absence of signs of active bacterial infection; elevated CRP; a higher risk for mortality at blood tests. | NA |
| [1] | Italy | NA | One center | Severe COVID-19 patients | Hyper inflammation (CRP ≥100mg/L, Ferritin≥900ng/mL, LDH≥220U/L) | A first dose of 400mg iv. A second 400mg was administered after 24h if worsening |
| [29] | USA | 2020-3-1 to 2020-4-22 | 13 centers | COVID-19 patients who needed support in ICU | With evidence of ARDS on mechanical ventilation, or worsening oxygenation with high oxygen requirements; symptoms had to be present for 7 days | 98% 400mg flat dosing, 1% 8mg/kg, 1% other doses |
| [49] | France | NA | One center | Severe COVID-19 patients (SP_O2_≤96% with O_2_supoort≥6L/min) without mechanical ventilation | The indication depended on an attending physician | Single-dose: 400mg |
| [28] | France | 2020-4-6 to 2020-4-21 | One center | Patients with severe COVID-19 | Extensive bilateral lesions in the lungs; increased CRP level; clinical aggravation; Severe respiratory insufficiency needing oxygen supplementation | 600mg for patients<100kg; 800mg for those>100kg |
| [31] | Italy | From 2020-2 (Total)  2020-3-14 to 2020-3-27 (Tocilizumab) | One center | Patients with COVID-19 | CRP>5mg/dl, PCT<0.5ng/ml, PF ratio<300, ALT<500U/L | 8 mg/kg (up to a maximum 800mg per dose); repeated after 12h if no side effects |
| [30] | Italy | Since 2020-2-26 | One Center | RR≥30 beats/min; peripheral capillary oxygen saturation≤93; PaO2/FiO2≤300mmHg | Within 4 days from hospital admission | 53% 400mg iv, 43.5% 324mg; 3.5% 800mg iv |
| [36] | Spain | 2020-3-10 to 2020-4-15 | One Center | Severe COVID-19 | NA | NA |
| [35] | Italy | 2020-2-21 to 2020-3-24 | 3 centers | Severe COVID-19 | RR≥30 beats/min; peripheral capillary oxygen saturation≤93; PaO2/FiO2≤300mmHg | 8mg/kg iv Q12H or subcutaneously at 324mg in total |
| [33] | Sweden | 2020-3-11 to 2020-4-15 | One center | Severe COVID-19 | At the discretion of the attending physician and required consultation of ≥ 2 infectious disease specialists and the fulfillment of criteria of respiratory and inflammatory parameters | Single dose at 8mg/kg |
| [34] | Spain | 2020-2-24 to 2020-3-23 | One center | Severe to critical COVID-19 | Measuring IL-6 serum levels to decide treatment with tocilizumab (>40pg/mL) | iv. 8mg/kg (maximum 800mg) followed by a second one after 12h |
| [38] | USA | 2020-3-13 to 2020-4-19 | One center | Oxygen saturation≤94% | Elevated inflammatory biomarkers (CRP>3g/dl or ferritin>400ng/ml), concern for clinical deterioration | Single dose iv. 8mg/kg |
| [40] | France | 2020-4-1 to 2020-5-11(Tocilizumab)  2020-3-1 to 2020-3-24(Control) | One center | COVID-19 Patients in critical condition | Period since symptoms onset ≥ 5 days, oxygen therapy ≥ 4L/min, ≥ 25% of lung damages, ≥ 2 parameters of inflammation or biological markers of mortality | 8mg/kg per dose, 1 or2 doses |
| [41] | Spain | 2020-1-31 to 2020-4-23 | 17 Centers | Patients with COVID-19 | NA | NA |
| [42] | Spain | 2020-3-10 to 2020-4-17 | One center | Patients with COVID-19 | Based on decreased oxygen saturation levels and increased RR, increased inflammatory biomarkers | At a dose of 600mg IV if weight was ≥75kg or 400mg when weight<75kg |
| [43] | Italy | 2020-3-10 to 2020-4-14 | One center | Patients with severe COVID-19 with worsening ARF | Serum CRP and IL-6 levels at admission were elevated | 8mg/kg-max 800mg |
| [50] | Italy | 2020-3-13 to 2020-4-3 (Tocilizumab)  Different time (Control) | One center | RR≥30 beats/min; peripheral capillary oxygen saturation≤93; PaO2/FiO2≤300mmHg | Presence of an active inflammatory status (CRP>1mg/dL, IL-6>40pg/mL, D-dimer>1.5mcg/mL, or ferritin>500ng/mL) | 8 mg/kg IV over 60min (maximum dose of 800mg); a second dose IV after 12 h |
| [52] | China | 2020-1 to 2020-2 | One center | NA | NA | NA |
| [48] | USA | 2020-3-8 to 2020-4-25 | One center | Patients with severe COVID-19 | NA | NA |
| [32] | Italy | After 2020-3-13 (Tocilizumab)  2020-2-26 to 2020-3-13 (Control) | One center | Patients with severe COVID-19 at early stage of respiratory failure | NA | 400mg iv or 324mg subcutaneous |
| [58] | Spain | 2020-2-2 to 2020-3-31 | 60 centers | Patients with COVID-19 with at least one clinical criterion and one laboratory criterion suggestive of a hyperinflammatory state | Ferritin>2000ng/mL or increase >1000ng/mL since admission, D-dimmer>1500ug/mL, IL>50pg/mL | NA |
| [46] | Italy | 2020-3-28 to 2020-4-21 | One center | ≥20% lung parenchyma on chest CT, hyperinflammation, hypoxemia | CRP≥20mg/dL, had no contraindications to treatment | 324mg given as 2 injection |
| [47] | Italy | 2020-3-28 to 2020-4-21 (Tocilizumab)  2020-3-7 to 2020-3-27(Control) | One center | COVID-19 patients with hyperinflammation, no hypoxemia | Bilateral pneumonia, hyper-inflammation (CRP≥20mg/dL), no hypoxaemia, and no contraindications; within 48h of admission. | Administered subcutaneously in two simultaneous 162mg |
| [45] | USA | 2020-3-13 to 2020-4-21 | One centers | Patients with solid organ transplant recipients and COVID-19 | ≥ 7 days of symptoms, progressive respiratory distress and rising levels of inflammatory markers (CRP, ferritin or IL-6) | 4-8mg/kg (maximum 800mg) |
| [44] | USA | 2020-3-10 to 2020-4-10 | One center | Patients with COVID-19 | Within 48h of admission; decreased WBC; elevated inflammatory markers (ferritin, LDH, PCT, ESR, CRP) | 4-8mg/kg (maximum 800mg) as a single dose or repeated dose in 12h in patients who remain febrile |
| [39] | USA | 2020-3-1 to 2020-4-27 | One center | Critically ill COVID-19 patients | Progressive clinical deterioration and elevated inflammatory markers | A dose of 400mg of tocilizumab IV |
| [37] | USA | 2020-3-4 to 2020-5-10 | Multi-centers | Critically ill COVID-19 patients | Tocilizumab was treated within 2 days of ICU admission | NA |
| [51] | USA | 2020-3-9 to 2020-4-20 | One center | Patients with moderate to severe COVID-19 | With rapid respiratory deterioration and evidence of hyper-inflammation | 4-8mg/kg, with a suggested dose of 400mg |
| [57] | USA | 2020-3-9 to 2020-5-7 | One center | Severe COVID-19 | Be administered within 24h of intubation | NA |
| [26] | Italy | 2020-3-31 to 2020-6-11 | 24 centers | Severe COVID-19 | PaO2/FiO2 200-300mmHg, temperature>38 during the last 2 days, CRP>10mg/dL | 8mg/kg (maximum 800mg), followed by a second dose after 12h |
| [27] | France | 2020-3-21 to 2020-4-18 | Multi-center | Patients with moderate, severe, or critical pneumonia | NA | 8mg/kg IV on day 1 and day 3. |
| [25] | Italy | 2020-4-20 to 2020-6-15 | 7 centers | ≥2 of the following: fever, pulmonary infiltrates, or need for supplemental oxygen in other to maintain an oxygen saturation >92% | CRP>50mg/L, ferritin >500ng/mL, D-dimmer>1000ng/mL, LDH>250U/L | Tocilizumab with 8mg/kg IV |

COVID-19: 2019-novel coronavirus disease; USA: the United States of American; NA: not available; IV: intravenously; CRP: C-reactive protein, LDH: lactate dehydrogenase; ARDS: acute respiratory distress syndrome; PCT: procalcitonin; ALT: alanine aminotransferase; ESR: estimated sedimentation rate
